# Determination of lindane in surface water samples and its degradation by hydrogen peroxide and persulfate assisted TiO_2_-based photocatalysis†

**DOI:** 10.1039/d3ra03610c

**Published:** 2023-07-10

**Authors:** Sanaullah Khan, Javed Ali Khan, Noor S. Shah, Murtaza Sayed, Muhammad Ateeq, Sabah Ansar, Grzegorz Boczkaj, Umar Farooq

**Affiliations:** a Departmen of Chemistry, Women University Swabi 23430 Pakistan; b Department of Chemistry, Abdul Wali Khan University Mardan Mardan 23200 Pakistan javedkhan@awkum.edu.pk khanjaved2381@gmail.com +92-937-542189 +92-937-929122; c Department of Environmental Sciences, COMSATS University Islamabad, Vehari Campus 61100 Pakistan; d Radiation Chemistry Laboratory, National Centre of Excellence in Physical Chemistry, University of Peshawar Peshawar 25120 Pakistan; e Department of Clinical Laboratory Sciences, College of Applied Medical Sciences, King Saud University P.O. Box 10219 Riyadh 11433 Saudi Arabia; f Department of Sanitary Engineering, Faculty of Civil and Environmental Engineering, Gdansk University of Technology G. Narutowicza St. 11/12 80-233 Gdansk Poland; g EkoTech Center, Gdansk University of Technology G. Narutowicza St. 11/12 80-233 Gdansk Poland; h Department of Chemistry, COMSATS University Islamabad, Abbottabad-Campus 22060 Abbottabad Pakistan; i Beijing National Laboratory for Molecular Sciences, State Key Laboratory of Molecular Reaction Dynamics, Institute of Chemistry, Chinese Academy of Sciences Beijing 100190 China

## Abstract

Organochlorine pesticides (OCPs) have been used extensively as insecticides and herbicides. This study investigates the occurrence of lindane in surface water from the Peshawar valley (*i.e.*, Peshawar, Charsadda, Nowshera, Mardan and Swabi districts of Khyber Pakhtunkhwa, Pakistan). Out of 75 samples tested (*i.e.*, 15 samples from each district), 13 samples (including 2 from Peshawar, 3 from Charsadda, 4 from Nowshera, 1 from Mardan, and 3 from Swabi) are found to be contaminated with lindane. Overall, the detection frequency is 17.3%. The maximum concentration of lindane is detected in a water sample from Nowshera and found to be 2.60 μg L^−1^. Furthermore, the degradation of lindane in the water sample from Nowshera, containing the maximum concentration, is investigated by simulated solar-light/TiO_2_ (solar/TiO_2_), solar/H_2_O_2_/TiO_2_ and solar/persulfate/TiO_2_ photocatalysis. The degradation of lindane by solar/TiO_2_ photocatalysis is 25.77% after 10 h of irradiation. The efficiency of the solar/TiO_2_ process is significantly increased in the presence of 500 μM H_2_O_2_ and 500 μM persulfate (PS) (separately), represented by 93.85 and 100.00% lindane removal, respectively. The degradation efficiency of lindane is lower in natural water samples as compared to Milli-Q water, attributed to water matrix effect. Moreover, the identification of degradation products (DPs) shows that lindane follows similar degradation pathways in natural water samples as the one in Milli-Q water. The results show that the occurrence of lindane in surface waters of Peshawar valley is a matter of great concern for human beings and the environment. Interestingly, H_2_O_2_ and PS assisted solar/TiO_2_ photocatalysis is an effective method for the removal of lindane from natural water.

## Abstract 2

Detection of lindane in surface waters and its degradation by hydroxyl and sulfate radical-based photocatalysis: degradation products formation and toxicity evaluation.

## Graphical abstract

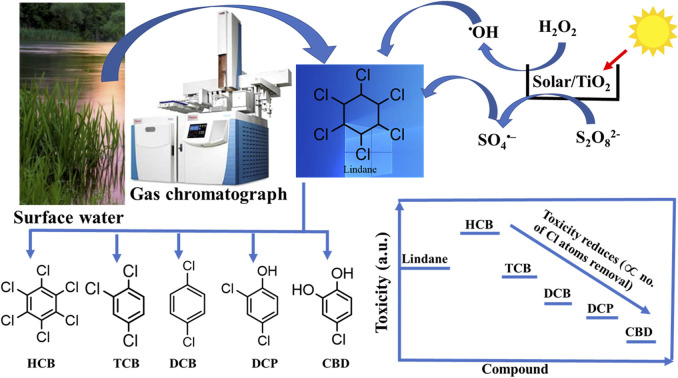

## 1. Introduction

Pesticides – synthesized organic compounds – have dramatically promoted agricultural production worldwide *via* pest control.^1^ Besides, pesticides are used in a number of non-agricultural applications such as livestock, forest industry, sprays and domestic products.^2^ Worldwide, about 2.5 million tons of pesticides are used annually and alarmingly, this number increases with the passage of time.^3^ Being an agricultural country, the largest source of economy in Pakistan is the agriculture sector. For instance, in 2004, about 23.3% of the GDP and 42.1% of the labor force in Pakistan come from the agriculture sector.^4^ A large quantity of pesticides is regularly used in Pakistan, aiming at an increased agricultural production – demanded for the high population of the country. Due to widespread application, pesticide residues may enter the water environment, mainly *via* direct runoff, air deposition, leaching, long-range transportation, equipment washings, disposal of containers, and as effluents from the manufacturing industries.^5–7^ A high frequency of pesticide residues was found in surface and ground waters as well as in the shallow drinking wells, especially in agriculturally developed areas of Pakistan.^3,7–10^ Moreover, a large number of different pesticides were frequently detected in river waters of Pakistan, such as river Kabul, Ravi and Indus.^11–13^

Among various classes of pesticides, organochlorine pesticides (OCPs) are used in relatively high quantity due to their higher efficacy. In the present study, lindane was selected as a model pollutant and representative of OCPs. Lindane (also called hexachlorocyclohexane, γ-HCH) – an organochlorine pesticide – has been extensively used against a large number of pests in the last several decades.^14^ Lindane has also been widely used in livestock, horticulture, forestry, pharmaceuticals and personal care products.^2^ It is used as a biocide for enhancing durability of indoor materials such as wood, leather, wool and cotton.^15^ Presently, it is used as an active ingredient in a variety of personal hygiene products such as lotions, creams and shampoos for the treatment of lice and scabies.^16,17^ Lindane is recognized as a highly chlorinated organic pollutant in the environment, and is considered as a possible neurotoxin, endocrine disruptor and a possible human carcinogen.^2,17–19^ Due to the excessive applications of lindane for multiple purposes during the last several decades, followed by its persistency as well as long-range transportation, it was frequently detected in the water environments in many regions around the world, including Europe,^20^ USA,^21^ Africa,^22^ China,^23^ India^24^ and Pakistan.^7^ Lindane was found in the far away regions of the world as well, *i.e.*, Arctic and Antarctica.^25^ The large-scale applications, high persistency, bioaccumulation and magnification tendency as well as high toxicity especially to the non-target species are some of the major issues concerned with the presence of lindane in the aquatic environment. Considering the toxic nature of lindane, a thorough investigation of its distribution in the water environment is of immense importance for environmental sustainability and public health welfare. Meanwhile, there is an urgent need for the development of environmentally friendly and sustainable methods for the removal of organochlorine pesticides from water. In this regard, advanced oxidation processes (AOPs), particularly TiO_2_ photocatalysis is a promising technology.^26–28^

This study investigated the determination of lindane in surface water of Peshawar valley, comprising of five districts, *i.e.*, Peshawar, Nowshera, Charsadda, Mardan and Swabi, of Khyber Pakhtunkhwa province, Pakistan. The validation of the extraction and analysis methods was done by using precision, accuracy, relative recovery, limit of detection (LOD) and limit of quantification (LOQ). Meanwhile, the removal of lindane from real water by an environmentally friendly and sustainable advanced oxidation process, *i.e.*, solar light assisted-TiO_2_ photocatalysis was investigated. The effect of hydrogen peroxide (H_2_O_2_) and persulfate (PS, S_2_O_8_^2−^) on the efficiency of solar/TiO_2_ photocatalysis in the natural water was investigated. Moreover, the radical scavenger tests were performed to investigate the role of various reactive species towards degradation of lindane by solar/TiO_2_ photocatalysis. Finally, the degradation products (DPs) of lindane by solar/H_2_O_2_/TiO_2_ and solar/PS/TiO_2_ photocatalysis in the natural water sample of Nowshera was identified by gas chromatography/mass spectrometry (GC-MS).

## 2. Materials and methods

### 2.1 Materials

Lindane (C_6_H_6_Cl_6_, 97%), sodium persulfate and titanium(iv) isopropoxide (TTIP, 97%) were purchased from Sigma-Aldrich. Hydrogen peroxide (H_2_O_2_, 50% v/v) was obtained from Fisher Scientific. Commercial grade plastic bottles (100 mL) were used for the collection of water samples. All the chemicals were used as received. Milli-Q water (resistivity = 18.2 MΩ cm) was used for preparation of standard solutions of lindane while obtaining the calibration curve.

### 2.2 Synthesis of TiO_2_ films

The synthesis of TiO_2_ photocatalyst was performed using a sol–gel method. The preparation of film types of TiO_2_ photocatalyst was carried out by a dip-coating technique using a TiO_2_ solution. The synthesis method and characterizations of TiO_2_ film can be seen in our previous paper.^29^

### 2.3 Sample collection

The water samples were collected from different sites of district Peshawar, Charsadda, Nowshera, Mardan and Swabi of Khyber Pakhtunkhwa, Pakistan.

Peshawar is the capital of Khyber Pakhtunkhwa province of Pakistan. In Pakistan, it is the sixth most populated city. It is the habitant of over 2.3 million people. People use water of (tube)wells for drinking and other domestic purposes. For irrigation purposes, mainly canal and river water are used. In Peshawar, the samples were collected from Taru Jabba, Tarnab, Chamkani, Wadpagga, Bakhshi Pull, Mathra, Tahkal, Pishtakhara, Saddar, Palosi, Nasir Bagh, Industrial Estate Hayatabad, Karkhano, Bara and Badaber.

Charsadda – the eighty fifth-largest city of Pakistan – is located in the Peshawar Valley. Its population is over 1.6 million. It is about 29 km away from Peshawar and located at an altitude of 276 metres. In Charsadda, the main source of irrigation is water of Jindi, Kabul and Swat rivers. The samples were collected from Sardaryab, Gulabad, Sarwani, Tarka Kalay, Kandar, Dheri Sikander Khan, Ambadher, Mirchakai Kalay, Rajar, Fazalabad, Akbarabad, Sardheri, Manga Dargai, Qala Korona and Dab Banda.

Nowshera is the 9th largest city in Khyber Pakhtunkhwa and 78th in Pakistan. Located on Kabul River, Nowshera lies in the Peshawar Valley and is approximately 43 km east of Peshawar. About 17 095 acre land in Nowshera is irrigated by the river Kabul. District Nowshera covers a total area of 1748 km^2^ and the population is 1.518 million. Nowshera is an industrially developed area in Khyber Pakhtunkhwa and it has a number of industries, mainly located in the Industrial estate Nowshera, comprising of around 40 units, and acquiring 108 acres area. Particularly, a DDT factory was established in Nowshera (Amangarh) in 1963, which, however, was closed down in 1994. District Nowshera has a vast agricultural land as well, *e.g.*, Nowshera Kalan and Pabbi, the latter being agriculturally more developed than the former. In this study, the sampling area included Pabbi, Jallozai, Pashtun Garhi, Azakhel, Miskeen Khel, Nowshera Kalan, Amangarh, Kabul River, Khat Kalay, Cantt, Tuheed Abad, Islamabad Koruna, Risalpur, Turlandi and Akora Khattak.

Mardan – after Peshawar – is the second largest city of Khyber Pakhtunkhwa having 1632 square km area and 2.25 million population. In Pakistan, Mardan is one of the best agricultural areas. Due to its land suitability for cultivation of tobacco and sugar cane, Mardan is known as the land of tobacco and sugar cane. Major crops grown in Mardan are maize, rice, sugarcane, wheat, and mustard. Main source of irrigation is canal water however, tube wells are also used. In the present study, surface water samples were collected from Nawan Killi, Toru, Rashakai, Mohabat Abad, Ghalla Dher, Faqir Abad, Mayar, Gujar Garhi, Janday, Seri Bahlol, Saro Shah, Bakhshali, Charguli, Shankar and Rustam.

Swabi district is located on the bank of the river Indus, 70 km westward of Islamabad. The total area of district Swabi is 1550 km^2^ and the population is 1.625 million. District Swabi is one of agriculturally more developed areas of Khyber Pakhtunkhwa and is especially known for production of high-quality tobacco and maize. The major sources of irrigation water in district Swabi are the two main canals, *i.e.*, the upper Swat canal and the Stefa canal. The water samples were collected from Shewa, Dagai, Ayaub Khan Kalay, Yar Hussain, Gumbat, Dobian, Ismalia, Adina, Kalu Khan, Sadbar Abad, Serai, Ambar, Saleem Khan, Shera Ghund and Tarakai.

All the samples were stored at 5 °C in clean plastic bottles before analysis. Prior to the SPME, the samples were filtered through 0.45 μm filter paper in order to remove particulate matter, if any.

### 2.4 SPME extraction

Solid phase micro-extraction (SPME) technique developed by Pawliszyn^30^ is a solvent-free alternative for the extraction of organic compounds from their aqueous solution. SPME is an easy to use, time saving, and easily automated technique.^31^ It is increasingly used in environmental applications, especially for the analysis of volatile organic compounds (VOCs),^32^ halocarbons,^33^ pesticides,^34^ and polychlorinated benzenes (PCBs).^35^ For extraction of lindane from water samples, SPME technique was used. The SPME was based on employing a poly(dimethylsiloxan)-divinylbenzene (PDMS-DVB) fiber coating protected by a metallic needle and operated by CTC Analytics Combi PAL auto-sampler. The water samples were placed in 20 mL glass vials with magnetic crimp-top caps capped with silicones PTFE lined septa. The lindane was extracted from the samples by holding the SPME fiber in the glass vials for 2 min. After extraction, the SPME fiber was injected into the GC inlet for thermal desorption, holding time was 2 min. The SPME fiber was washed with Milli-Q water after each run by dip-injecting method to remove any contaminants.

### 2.5 Degradation experiment

**Fig. 1:**
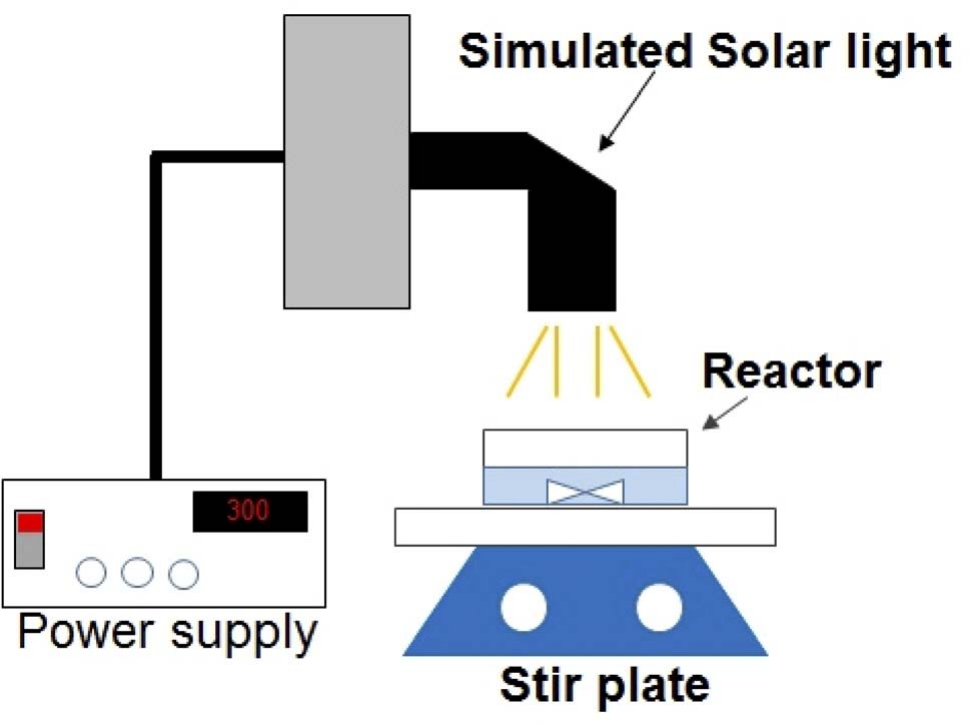
Schematic diagram of the photoreactor.

Degradation of lindane was performed in selected real water sample where its maximum concentration (*i.e.*, 2.60 μg L^−1^) was detected, *i.e.*, in water sample of Nowshera (Amangarh, N7). The physicochemical characteristics of this water sample are shown in Table S1, ESI.† The degradation experiments were carried out in a batch photoreactor, *i.e.*, a 100 mL beaker containing 50 mL of the water sample collected from Nowshera. The schematic diagram of the photoreactor is shown in Fig. 1. A TiO_2_ film was used as a photocatalyst. The reaction solution was illuminated by simulated solar light. The simulated solar light was achieved by a 300 W Xenon lamp (Newport, Oriel Instrument) with light irradiance (Ee) of 4.71 × 10^−2^ W cm^−2^, as measured by Newport broadband radiant power meter.

### 2.6 Lindane quantification and its degradation products identification

Gas chromatography-micro-cell electron capture detector (GC-μECD) is a modern analytical sophisticated technique frequently employed for the detection and quantification of organochlorine compounds in aqueous solution.^36^ The ECD is highly sensitive towards detecting the halogenated organic compounds at trace levels in environmental and biological samples.^37^

The samples were analyzed for quantification of lindane by using gas chromatography (Agilent 1200 N series, USA) coupled with micro-cell electron capture detector (GC-μECD) in a splitless mode. For separation purposes, HP-5 capillary column (30 m × 0.25 mm I.D. and 0.25 μm film thickness) was used. The programming of oven temperature was: 50 °C (2 min hold) to 150 °C at increasing rate of 10 °C min^−1^ (3 min hold), and then further to 250 °C at increasing rate of 20 °C min^−1^ (5 min hold). The GC injector and detector temperatures were set at 220 and 350 °C, respectively. Ultrapure N_2_ gas at a flow rate of 1.5 mL min^−1^ was used as a carrier gas.

Degradation products (DPs) of lindane were identified by gas chromatography/mass spectrometry (GC-MS; Agilent 6890) having an HP-5MS (5% phenyl methylsiloxane) capillary column (30 m × 0.25 μm). The temperature of the MS source and quadrapole was set at 230 and 150 °C, respectively. Electron impact ionization mode (EI^+^) set at 70 eV was used for obtaining mass spectra of the DPs. The *m*/*z* range was set from 50 to 550. DPs were identified by comparison to online NIST mass spectral library installed in the GC/MS. The rest of the conditions were same as of the GC-μECD.

### 2.7 Method validation

Calibration of GC-μECD was made using standard lindane solutions at seven different concentrations, *i.e.*, 0.05, 0.10, 0.50, 1.00, 3.00, 6.00, and 10.0 μg L^−1^, by plotting peak area (a.u) *versus* concentration. The concentration of lindane in the water samples was determined by using calibration curve (*via* least square method using straight line equation obtained from the calibration plot).

Limit of detection (LOD), *i.e.*, the minimum concentration of an analyte that can be differentiated from the blank or background signal with a certain limit of confidence,^38^ was determined at a signal to noise (S/N) ratio of 3. The limit of quantification (LOQ), *i.e.*, the smallest concentration of analyte that can be determined quantitatively with certainty^38^ was determined at S/N ratio of 10.

The precision, accuracy and relative recoveries of the optimized method was investigated at three concentration levels (2.5, 5.0 and 10.0 μg L^−1^) in a triplicate manner. The precision was measured in terms of relative standard deviation (RSD). The intra-day precision and accuracy were determined by analyzing the lindane samples at mentioned concentrations (in triplicate) on the same day, while the inter-day precision and accuracy were obtained by analyzing the samples on three different days. The accuracy of the optimized method was calculated by using the following equation:1
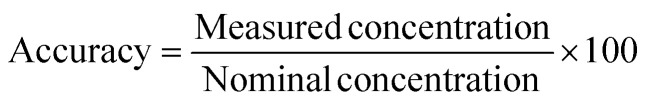


To assess the effect of matrices on the concentration of lindane in the field water, the relative recoveries were obtained for a set of six samples, prepared in the field water. Of the six samples used for relative recoveries, three samples did not contain any detectable quantity of lindane, while the remaining three samples do contain some detectable amount of lindane. The relative recoveries were determined by using eqn (1), but in this case lindane solution was prepared in field water rather than Milli-Q water, as in the case of accuracy measurement.

### 2.8 Toxicity evaluation

To examine the toxicity of lindane and its detected DPs, the acute (LC_50_/EC_50_) and chronic toxicities (ChV) were calculated using the Ecological Structure Activity Relationship (ECOSAR) program – an effective-prediction tool of the substances' toxicities towards three aquatic organisms (*i.e.*, fish, daphnia and green algae).^39^ The acute toxicity is represented by LC_50_ and EC_50_. LC_50_ is the concentration of toxicant which results in the death of 50% fish and daphnia after 96 and 48 h contact times, respectively.^40^ Similarly, EC_50_ is the concentration of toxicant which results in the 50% growth inhibition of green algae after 96 h contact time.^40^ According to the European Union criteria, a compound is considered as: (i) “not harmful” if LC_50_/EC_50_ > 100 mg L^−1^, (ii) “harmful” if LC_50_/EC_50_ = 10 to 100 mg L^−1^, (iii) “toxic” if LC_50_/EC_50_ = 1 to 10 mg L^−1^, and (iv) “very toxic” if LC_50_/EC_50_ < 1 mg L^−1^.^41^ Similarly, in accordance of the Chinese hazard evaluation criteria, a compound could be classified as: (i) “not harmful” if ChV > 10 mg L^−1^, (ii) “harmful” if ChV = 1 to 10 mg L^−1^, (iii) “toxic” if ChV = 0.1 to 1 mg L^−1^, and (iv) “very toxic” if ChV < 0.1 mg L^−1^.^41^

## 3. Results and discussion

### 3.1 Method validation

A suitable method was developed for the analysis of lindane using GC-μECD according to the parameters shown in Section 2.6. The detector showed a linear response with *R*^2^ = 0.997 in the concentration range of 0.05 to 10.0 μg L^−1^ as shown in Table S2, ESI.† The LOD and LOQ determined at S/N of 3 and 10 were found to be 0.008 μg L^−1^ and 0.037 μg L^−1^, respectively (Table S2†). As shown in Table 1, very precise, accurate and more reproducible results were obtained under both intra-day and inter-day conditions.

**Table 1:** Intra-day and inter-day precision, accuracy and reproducibility of the optimized method

Analyte	Nominal concentration (μg L^−1^)	Intra-day response			Inter-day response		
		Measured concentration (mean ± SD)	Precision (RSD)	Accuracy (%)	Measured concentration (mean ± SD)	Precision (RSD)	Accuracy (%)
Lindane	2.5	2.64 ± 0.27	10.29	105.37	2.37 ± 0.28	11.91	94.78
	5.0	4.82 ± 0.25	5.15	96.36	5.03 ± 0.26	5.12	100.55
	10.0	9.20 ± 0.71	7.68	92.00	9.27 ± 0.39	4.21	92.70

The relative recoveries of lindane by SPME-GC-μECD method in six different field water samples ranged from 83 to 114% (Table 2). The good RSD (%) values were also achieved for lindane analysis by the stated method as shown in Table 2.

**Table 2:** Relative recoveries (%) and RSD (%) of lindane by SPME-GC-ECD method in six different field water samples^a^

Lindane concentration (μg L^−1^)	Field water sample											
	P1		C1		N1		N8		M1		S1	
	Relative recovery	RSD	Relative recovery	RSD	Relative recovery	RSD	Relative recovery	RSD	Relative recovery	RSD	Relative recovery	RSD
2.5	88.18	8.98	110.57	7.87	88.58	13.18	114.16	2.64	103.37	16.04	99.57	17.48
5.0	98.46	9.16	101.05	11.28	95.66	14.84	103.45	8.99	96.56	3.11	92.06	7.77
10.0	83.31	9.78	88.40	8.09	99.69	7.14	99.15	2.56	88.05	5.17	100.34	5.31

a P1 (Taru Jabba, Peshawar); C1 (Sardaryab, Charsadda); N1 (Pabbi, Nowshera); N8 (Kabul River, Nowshera); M1 (Nawan Killi, Mardan); S1 (Shewa, Swabi).

### 3.2 Determination of lindane in collected water samples

Table 3 show the concentration of lindane in water samples of district Peshawar, Charsadda, Nowshera, Mardan and Swabi. In Peshawar, only 2 samples (out of 15) were contaminated with lindane, corresponding to a detection frequency of 13.3%. The lindane concentration varied in the range of 0 (*i.e.*, non-detectable, ND) to 0.63 μg L^−1^. Detection frequency was found to be 20.0% for Charsadda water samples where lindane was detected in 3 out 15 samples. Here, the maximum concentration was found to be 1.01 μg L^−1^ in water collected from Mirchakai Kalay. As for as district Nowshera is concerned, here lindane was found in 4 samples corresponding to detection frequency of 26.7%. This is the maximum detection frequency among the studied districts. The maximum concentration detected in water samples of Nowshera district was 2.60 μg L^−1^. This is the maximum concentration found among the tested water samples of all the five districts. Luckily, only one water sample collected from Mardan district contains lindane at a concentration level of 0.22 μg L^−1^. However, in water samples of district Swabi, 3 samples were observed to have lindane with a maximum concentration of 0.28 μg L^−1^.

**Table 3:** Detected concentration of lindane in surface waters of various district

Sample ID	Collection spot	Concentration (μg L^−1^)	Sample ID	Collection spot	Concentration (μg L^−1^)
**Peshawar**					
P1	Taru Jabba	ND	P9	Saddar	ND
P2	Tarnab	0.63	P10	Palosi	ND
P3	Chamkani	ND	P11	Nasir Bagh	ND
P4	Wadpagga	ND	P12	Hayatabad	ND
P5	Bakhshi Pull	ND	P13	Karkhano	ND
P6	Mathra	0.32	P14	Bara	ND
P7	Tahkal	ND	P15	Badaber	ND
P8	Pishtakhara	ND			

**Charsadda**					
C1	Sardaryab	0.25	C9	Rajar	ND
C2	Gulabad	ND	C10	Fazalabad	ND
C3	Sarwani	ND	C11	Akbarabad	ND
C4	Tarka Kalay	ND	C12	Sardheri	ND
C5	Kandar	ND	C13	Manga Dargai	ND
C6	Dheri Sikander Khan	0.56	C14	Qala Korona	ND
C7	Ambadher	ND	C15	Dab Banda	ND
C8	Mirchakai Kalay	1.01			

**Nowshera**					
N1	Pabbi	ND	N9	Khat Kalay	ND
N2	Jallozai	0.71	N10	Cantt	ND
N3	Pashtun Garhi	ND	N11	Tuheed Abad	0.64
N4	Azakhel	ND	N12	Islamabad Koruna	ND
N5	Miskeen Khel	ND	N13	Risalpur	ND
N6	Nowshera Kalan	ND	N14	Turlandi	ND
N7	Amangarh	2.60	N15	Akora Khattak	ND
N8	Kabul River	0.32			

**Mardan**					
M1	Nawan Killi	ND	M9	Janday	ND
M2	Toru	ND	M10	Seri Bahlol	ND
M3	Rashakai	ND	M11	Saro Shah	ND
M4	Mohabat Abad	ND	M12	Bakhshali	ND
M5	Ghalla Dher	ND	M13	Charguli	ND
M6	Faqir Abad	ND	M14	Shankar	ND
M7	Mayar	ND	M15	Rustam	0.22
M8	Gujar Garhi	ND			

**Swabi**					
S1	Shewa	0.14	S9	Kalu Khan	ND
S2	Dagai	ND	S10	Sadbar Abad	ND
S3	Ayaub Khan Kalay	ND	S11	Serai	ND
S4	Yar Hussain	ND	S12	Ambar	ND
S5	Gumbat	ND	S13	Saleem Khan	0.28
S6	Dobian	ND	S14	Shera Ghund	ND
S7	Ismalia	ND	S15	Tarakai	0.19
S8	Adina	ND			

In Pakistan, pesticides are mostly used for agricultural purposes. Thus, the main cause of lindane in surface water could possibly be the run-off waters from agricultural fields. An outlook of pesticides consumption in Pakistan during 1997–2006 is provided in Table S3, ESI.†^42^ Other uses of lindane include: as a biocide agent for making wood, leather, wool and cotton durable, and as an anti-scabies and anti-lice agent in personal hygiene products such as lotions, creams and shampoos. Consequently, the effluents of wood, paper and pulp, leather and cotton industries may possibly contribute to the entrance of lindane in surface waters. Besides, domestic wash-off and drainage waters can have a minor contribution to the presence of lindane in water bodies if the lotions, creams and shampoos used in homes contain lindane as an active ingredient. In fact, due to the wide range applications of lindane, there could be numerous causes of lindane entrance into water bodies. Because of its persistent and non-biodegradable nature, lindane can accumulate in surface waters. Thus, minor contributions from several sources can lead to lindane concentration in surface waters at detectable and quantifiable level. As a result, 17.3% water samples were found to have lindane at quantifiable level. Among the five districts, the detection frequency of lindane in surface waters follows the order: Nowshera > Charsadda = Swabi > Peshawar > Mardan with detection frequency of 26.7, 20.0, 20.0, 13.3 and 6.7%, respectively. Moreover, the highest concentration of lindane in 75 tested water samples was also detected in Nowshera where the surface water of Amangarh has 2.60 μg L^−1^ of lindane. This concentration is beyond the maximum acceptable level (MAL) for a single pesticide in surface water, *i.e.*, 1.0 μg L^−1^, according to parametric guideline values of European Union.^43^ Of note, the European Union has set the maximum permissible limit in drinking water as 0.1 μg L^−1^ for an individual pesticide and 0.5 μg L^−1^ for total pesticides.^44^

A DDT factory was established in Amangarh, district Nowshera, which annually produced 700 000 kg of technical grade dichlorodiphenyltrichloroethane (DDT) from 1963 to 1994.^45^ This DDT factory was abandoned after the ban on production and uses of persistent organic pollutants (POPs) by the Stockholm convention 2001.^46^ Even after officially shut down, the factory was kept operational for several years.^45^ Though the factory has been demolished, higher concentration of DDT has been detected in soil and water samples several years after the factory closure.^47^ The inappropriate dumping of the DDT and its raw materials in the factory site will keep contaminated the soil and water with DDT in the vicinity of the factory. Though the DDT factory was producing only DDT, the presence of lindane in the water sample of Amangarh could be due to the formation of lindane as a byproduct during the synthesis of DDT. Another possibility is the large-scale applications of pesticides in the surrounding area for agricultural purposes. Irrespective of the sources of lindane in the surface waters, its presence in water bodies could pose serious threats to human beings and other living animals. Hence, development of cheap and effective remediation technologies is urgently needed to tackle this problem efficiently.

### 3.3 Photocatalytic degradation of lindane in real water

**Fig. 2:**
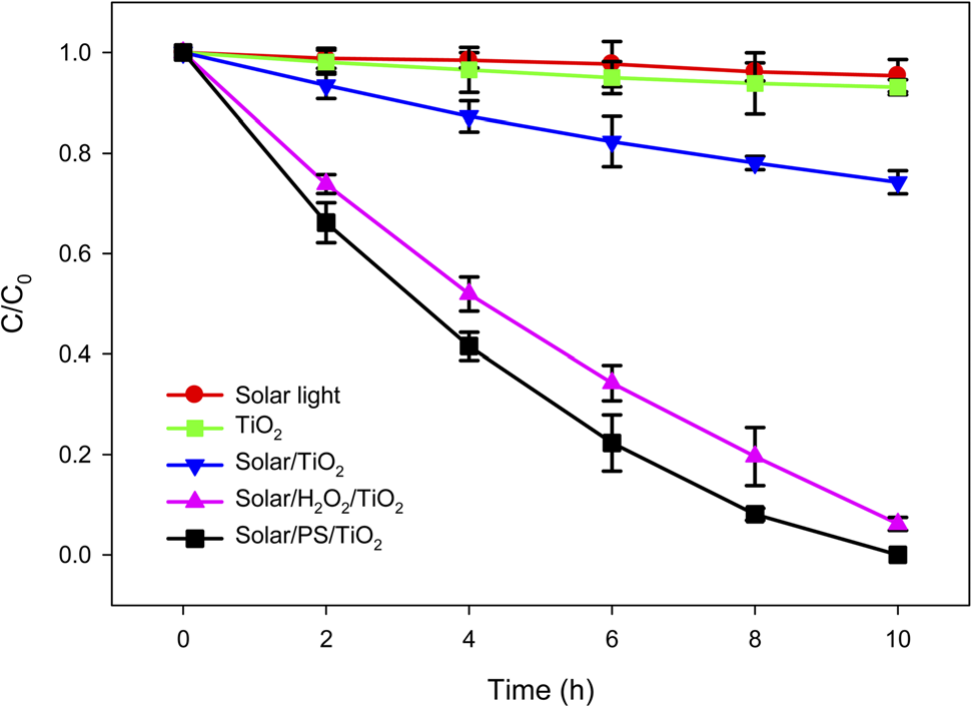
Photocatalytic degradation of lindane. Experimental conditions: [Lindane]_0_ = 2.60 μg L^−1^; [H_2_O_2_]_0_ = [PS]_0_ = 500 μM; Mass of TiO_2_ film = 9.02 mg; thickness of TiO_2_ film = 1.02 μm; area of TiO_2_ film = 3750 mm^2^; pH = 7.3.

The lindane concentration found in the real water (RW) sample of Amangarh (*i.e.*, [lindane] = 2.60 μg L^−1^), was the highest concentration of lindane in the studied region of Peshawar valley. This water sample was subjected to simulated solar light-assisted TiO_2_ photocatalysis (solar/TiO_2_) (Fig. 2). The results showed that solar/TiO_2_ process can achieve 25.77% of lindane degradation, at treatment time of 10 h, in RW sample of Amangarh (see the physicochemical properties of this water sample in Table S1, ESI†).

Activation of semiconductor photocatalysts such as TiO_2_ in aqueous solutions by light photons could lead to the generation of reactive species in the form of hydroxyl radicals (˙OH) and superoxide radical anions (O_2_˙^−^) as shown in reactions (2)–(5).^48–50^ These reactive species are generally responsible for the degradation of target organic pollutants such as lindane, in the present case.

2 TiO_2_ + *hν* → h_VB_^+^ (valence band hole) + e_CB_^−^ (conduction band electron)

3 e_CB_^−^ + O_2_ → O_2_˙^−^

4 h_VB_^+^ + H_2_O → ˙OH + H^+^

5 h_VB_^+^ + OH^−^ → ˙OH

Since lindane degradation/removal by solar light and TiO_2_ was only 4.6 and 6.9%, respectively, at 10 h of treatment (Fig. 2), ˙OH and O_2_˙^−^ could possibly contribute to the degradation of lindane by solar/TiO_2_ photo-catalysis to achieve 25.77% degradation in same treatment time. The degradation of lindane by ˙OH was previously reported in the literature using photo-Fenton process,^51^ photocatalysis^52^ and UV/H_2_O_2_.^53^

Since the solar/TiO_2_ photocatalytic degradation of lindane in Amangarh water was relatively low (25.77% degradation in 10 h), hydrogen peroxide (H_2_O_2_) and persulfate (PS, S_2_O_8_^2−^) was added (500 μM each one) – separately – to the solar/TiO_2_ system to accelerate the lindane degradation (Fig. 2). It can be seen that the degradation of lindane was increased from 25.77% to 93.85 and 100.00% in presence of H_2_O_2_ and PS, corresponding to an increase in *k*_obs_ from 0.0309 h^−1^ to 0.1891 and 0.2383 h^−1^, respectively. This increase in photo-catalytic degradation efficiency of lindane by H_2_O_2_ and PS was due to the production of additional ˙OH in solar/H_2_O_2_/TiO_2_ (reactions (6) and (7)) and SO_4_˙^−^ and ˙OH in solar/PS/TiO_2_ processes (reactions (8)–(10)).^54^ Both H_2_O_2_ and PS are good electron acceptors, thus, effectively scavenge the photogenerated electrons (e_CB_^−^), (reactions (6) and (8)), thus suppressing the recombination rate of h_VB_^+^ and e_CB_^−^, thereby enhancing the concentration of h_VB_^+^, subsequently increasing the concentration of ˙OH at the TiO_2_ surface.

6 H_2_O_2_ + e_CB_^−^ → ˙OH + HO^−^

7 H_2_O_2_ + *hv* → 2 ˙OH (*λ* = 254 nm, *ϕ* = 1.0)

8 S_2_O_8_^2−^ + e_CB_^−^ → SO_4_^2−^ + SO_4_˙^−^

9 S_2_O_8_^2−^ + *hv* → 2 SO_4_˙^−^ (*λ* = 254 nm, *ϕ* = 1.8)

10 SO_4_˙^−^ + H_2_O → SO_4_^2−^ + ˙OH + H^+^

Moreover, the results further indicated that PS has higher enhancement effect on the photo-catalytic removal efficiency of lindane as compared to H_2_O_2_. The higher removal of lindane by solar/PS/TiO_2_ as compared to by solar/H_2_O_2_/TiO_2_ could be attributed to: (i) higher quantum yield of SO_4_˙^−^ from S_2_O_8_^2−^ photolysis (*i.e.*, 1.8) as compared to the quantum yield of ˙OH from H_2_O_2_ photolysis (*i.e.*, 1.0); (ii) lower reactivity of natural organic matter (NOM) with SO_4_˙^−^ than ˙OH, suggesting lower scavenging efficiency of NOM for SO_4_˙^−^ as compared to ˙OH (reactions (11) and (12));^55^ (iii) higher redox potential of SO_4_˙^−^ than ˙OH under neutral conditions; and (iv) longer half-life of SO_4_˙^−^ than ˙OH (*i.e.*, 30–40 μs for SO_4_˙^−^ and < 1 μs for ˙OH).

11 NOM + ˙OH → Products *k* = 2.23 × 10^8^ L (mol C)^−1^ s^−1^

12 NOM + SO_4_˙^−^ → Products *k* = > 6 × 10^6^ L(mol C)^−1^ s^−1^

For better comparison, the results of solar/TiO_2_, solar/H_2_O_2_/TiO_2_ and solar/PS/TiO_2_ photocatalytic degradation of lindane in distilled water (DW) – performed in our previous study^29^ – has been provided in Table 4. Compared to DW results, the RW sample (Amangarh) showed lower removal efficiency of lindane by solar/TiO_2_ photocatalysis, attributed to the matrix effects, as indicated from the physico-chemical characteristics of the RW samples (Table S1, ESI†). Literature studies showed that water matrix might have neutral, inhibiting or enhancing effect on the degradation efficiency of organic pollutants using various advanced oxidation processes, depending on the nature and concentration of natural water constituents, as well as process and mechanism by which these constituents react within the system.^56^ The inhibiting effects due to the inorganic ions can be explained *via*: (i) scavenging/quenching of reactive radicals and/or h^+^, (ii) conversion of hydroxyl radicals to less reactive radicals, and (iii) adsorption of the ions to the active sites of the catalyst. While the enhancing effect of inorganic ions could be due to the formation of more reactive radicals.^56^ The presence of NOM and inorganic anions in RW of Amangarh (as shown in Table S1, ESI†) might have lowered the degradation efficiency of lindane, *via* scavenging of ˙OH/SO_4_˙^−^ and/or quenching of h^+^, according to reactions (11)–(14).^56–58^

13 X^*n*−^ + h^+^ → X˙^(*n*−1)−^

14 X^*n*−^ + ˙OH/SO_4_˙^−^ → X˙^(*n*−1)−^ + OH^−^/SO_4_^2−^/H_2_O

where X^*n*−^ represents Cl^−^, SO_4_^2−^, CO_3_^2−^, HCO_3_^−^ or NO_3_^−^, and X˙^(*n*−1)−^ represents Cl˙, SO_4_˙^−^, CO_3_˙^−^ or 
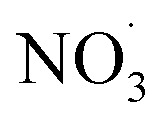
.

**Table 4:** Comparison of photo-catalytic degradation of lindane by solar/TiO_2_, solar/H_2_O_2_/TiO_2_ and solar/PS/TiO_2_ processes in Milli-Q water and natural water samples. Experimental conditions: [Lindane]_0_ = 1 μM in distilled water and 2.60 μg L^−1^ in natural water sample; [H_2_O_2_] = [PS] = 500 μM, mass of TiO_2_ film = 9.02 mg; thickness of TiO_2_ film = 1.02 μm; area of TiO_2_ film = 3750 mm^2^; pH 5.8 in distilled water and 7.3 in natural water sample

Type of water	Pseudo-first-order rate constant (*k*_obs_, h^−1^)			Reference
	Solar/TiO_2_	Solar/H_2_O_2_/TiO_2_	Solar/PS/TiO_2_	
Natural water sample of Amangarh	0.0309	0.1891	0.2383	This study
Distilled water	0.0423	0.2084	0.4948	29

Our results were consistent with the findings of Yuan *et al.*^59^ which showed that the degradation efficiency of sulfamethoxazole by TiO_2_ photocatalysis was reduced in the presence of Cl^−^ and SO_4_^2−^. Yap and Lim^60^ also found the degradation efficiency of bisphenol-A by solar light-assisted N–TiO_2_/activated carbon photocatalysis was decreased from 82 to 76, 77 and 76% in the presence of 100 mM of each Cl^−^, NO_3_^−^ and SO_4_^2−^, respectively, consistent with our results. Rehman *et al.*^61^ found that the degradation efficiency of diclofenac sodium by H_2_O_2_/catalyst process was decreased in the presence of NO_3_^−^ and SO_4_^2−^. The inhibiting effect of the inorganic ions was attributed to the scavenging of ˙OH. Also, the Cl^−^ might compete with organic compounds for the adsorption sites on the catalyst surface,^62^ and SO_4_^2−^ showed more adsorption capacity than Cl^−^.^63^ Owing to different water matrix constituents, the degradation efficiency of organic pollutants by AOPs could be varied in natural and real water systems, including river, surface, ground, deionized and wastewater. Salimi *et al.*^64^ showed that visible light-assisted D-g-C_3_N_4_–Bi_5_O_7_I photocatalytic degradation efficiency of metronidazole in deionized water was lowered from 89 to 85 and 77% in the tap and wastewater treatment plant effluent, respectively, attributed to radical scavenging by the water constituents such as NO_3_^−^ and SO_4_^2−^. Sayed *et al.*^65^ showed that the degradation efficiency of norfloxacin by gamma radiation based AOP was low in the surface and ground waters, attributed to radical scavenging effect of water constituents.

### 3.4 Relative contribution of different reactive species

**Fig. 3:**
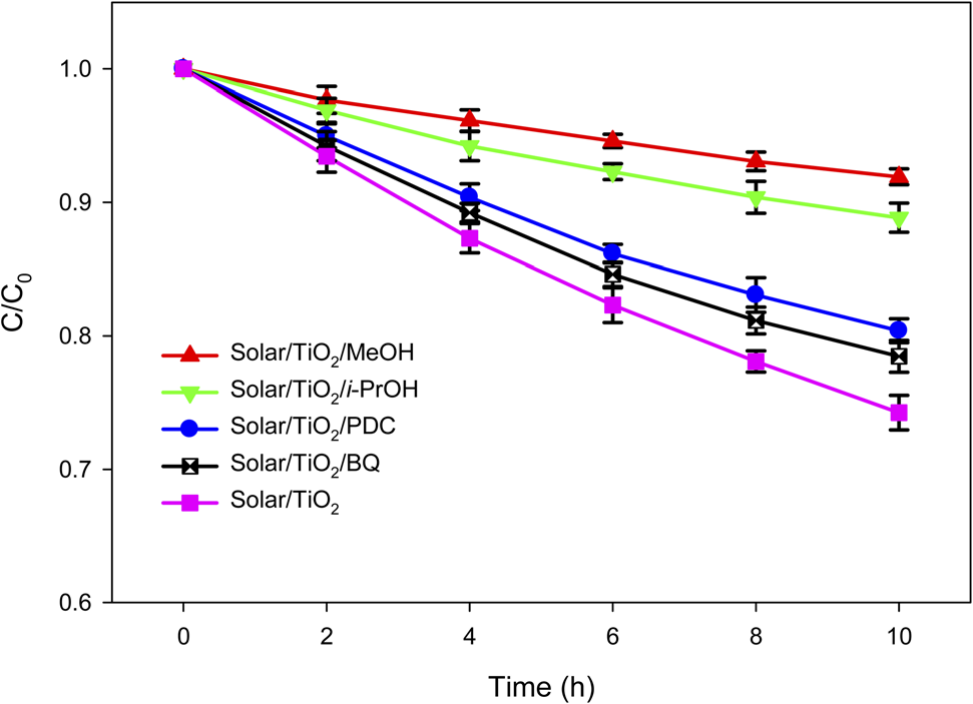
Effect of methanol (MeOH), iso-propanol (i-PrOH), benzoquinone (BQ) and potassium dichromate (PDC) on the photocatalytic degradation of lindane by solar/TiO_2_. Experimental conditions: [Lindane]_0_ = 2.60 μg L^−1^; [MeOH]_0_ = [*i*-PrOH]_0_ = [BQ]_0_ = 50 mM; [PDC]_0_ = 100 μM; mass of TiO_2_ film = 9.02 mg; thickness of TiO_2_ film = 1.02 μm; area of TiO_2_ film = 3750 mm^2^; pH = 7.3.

It has been reported that a number of different reactive species (RSs) such as ˙OH, O_2_˙^−^, h^+^, and e_CB_^−^ can be generated during the irradiation of TiO_2_ containing aqueous solution by solar or UV light.^66,67^ These RSs are then responsible for the degradation of organic pollutants. However, the contribution of these RSs towards organic compounds degradation depends on the nature of the target compound and the reaction system. To find out the relative contribution of these RSs towards lindane degradation by solar/TiO_2_ photo-catalysis, specific scavengers were used during the photocatalytic degradation of lindane (Fig. 3 and Table S4, ESI†).

Iso-propanol (i-PrOH), methanol (MeOH), *p*-benzoquinone (BQ) and potassium dichromate (PDC, K_2_Cr_2_O_7_) were used to quench ˙OH, h^+^ and ˙OH, O_2_˙^−^ and e_CB_^−^, respectively.^68,69^ The pseudo-first-order rate constants (*k*_obs_) were found to be 0.0309, 0.0126 and 0.0089, 0.0258 and 0.0230 h^−1^ in the presence of no scavenger, iso-propanol, methanol, BQ and PDC, respectively. The reduction in *k*_obs_ was calculated to be 71.20% (= (0.0309–0.0089)/0.0309 × 100) and 59.22% (= (0.0309–0.0126)/0.0309 × 100) in the presence of methanol and iso-propanol, respectively. Thus, the contribution of ˙OH towards lindane degradation was 59.22% and that from h^+^ was 11.97% (= 71.20–59.20%). The reduction in *k*_obs_ was calculated to be 16.50% (= (0.0309–0.0258)/0.0309 × 100) in presence of BQ, suggesting O_2_˙^−^ contributed 16.50% towards lindane degradation. Moreover, potassium dichromate reduced the *k*_obs_ by 25.57% (= (0.0309–0.0230)/0.0309 × 100). Although it can be concluded from these results that e_CB_^−^ contributed 25.57% towards lindane degradation, this is not direct contribution. The formation of O_2_˙^−^ in the reaction system was due to the reduction of dissolved O_2_ by e_CB_^−^ (reaction (3)), thus this 25.57% contribution also involved the contribution from O_2_˙^−^. In summary, the main RS in solar/TiO_2_ photo-catalysis was ˙OH towards lindane degradation.

### 3.5 Identification of degradation products

**Scheme 1:**
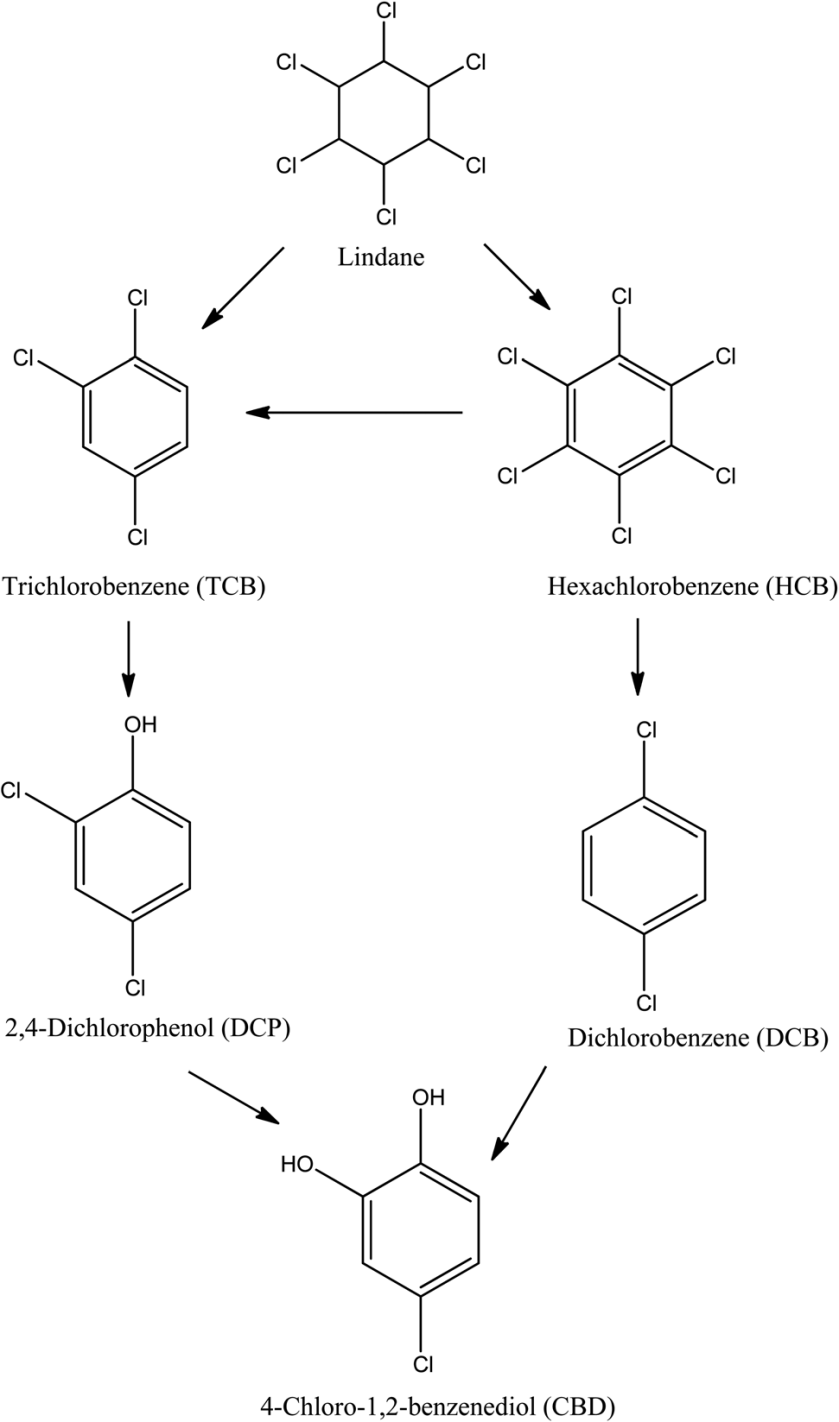
Proposed degradation pathway of lindane by solar/H_2_O_2_/TiO_2_ and solar/PS/TiO_2_ photocatalysis. All of these five DPs were detected in solar/H_2_O_2_/TiO_2_ while in solar/PS/TiO_2_ only three (HCB, TCB and DCB) were detected. Experimental conditions: [Lindane]_0_ = 500 μg L^−1^, [H_2_O_2_]_0_ = [PS]_0_ = 500 μM; mass of TiO_2_ film = 9.02 mg; thickness of TiO_2_ film = 1.02 μm; area of TiO_2_ film = 3750 mm^2^; pH = 7.3.

The solar/H_2_O_2_/TiO_2_ and solar/PS/TiO_2_ photocatalysis of lindane in real water of Amangarh was investigated for the identification of degradation products (DPs). Additional lindane was spiked into the real water sample to bring the concentration from 2.60 to 500 μg L^−1^ so that the DPs could be produced in easily detectable levels. Solar/H_2_O_2_/TiO_2_ resulted in the formation of five DPs namely: (i) hexachlorobenzene (HCB), (ii) trichlorobenzene (TCB), (iii) dichlorobenzene (DCB), (iv) 2,4-dichlorophenol (DCP), and (v) 4-chloro-1,2-benzenediol (CBD) (Scheme 1). Whereas, only the first three DPs, *i.e.*, (i) HCB, (ii) TCB and (iii) DCB, were detected in solar/PS/TiO_2_ photocatalysis. The formation of HCB occurs *via* dehydrogenation reaction (*i.e.*, removal of hydrogen), TCB and DCP produced *via* dehydrochlorination reaction (*i.e.*, removal of hydrogen chloride) and DCP and CBD formed *via* dechlorination–hydroxylation reactions. These results indicated that both ˙OH and SO_4_˙^−^ are capable of dehydrogenation and dechlorination reactions which led to the formation of HCB, TCB and DCB. Previously, hydrogen abstraction (removal of hydrogen) and dechlorination reactions of organic compounds by ˙OH and SO_4_˙^−^ have been reported.^70^ Although hydroxylation reaction by SO_4_˙^−^ has also been reported,^71,72^ the results of the present study – formation of DCP and CBD in solar/H_2_O_2_/TiO_2_ – indicated that hydroxylation reaction is the predominant property ˙OH. Moreover, it can also be concluded that there is relatively lower concentration of ˙OH in solar/PS/TiO_2_ (produced *via* reaction (10)) than in solar/H_2_O_2_/TiO_2_, otherwise DCP and CBD could be produced in solar/PS/TiO_2_. These detected DPs were identified in previous studies involving lindane degradation by ˙OH and/or SO_4_˙^−^ based AOPs.^52,54,71–73^ Interestingly, the results showed that although NOM and inorganic ions, *i.e.*, NO_3_^−^, SO_4_^2−^ and Cl^−^, had reduced the degradation efficiency of lindane, they had not affected the degradation mechanism of lindane.

### 3.6 Toxicity evaluation of lindane and its detected degradation products

**Fig. 4:**
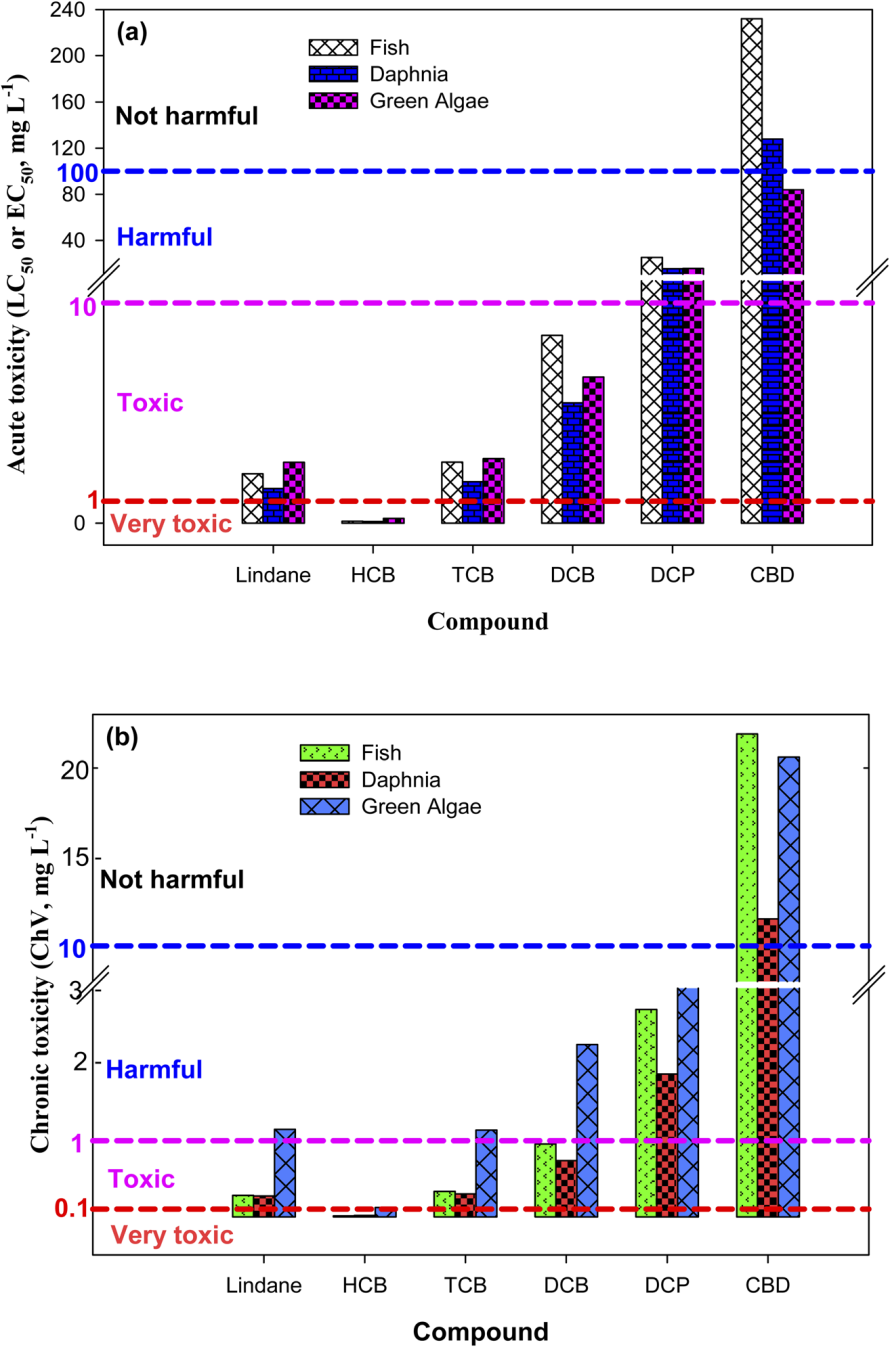
(a) Acute toxicity in terms of LC_50_ (fish and daphnia) and EC_50_ (green algae), and (b) chronic toxicity (ChV) of lindane and its detected degradation products towards fish, daphnia, and green algae in units of mg L^−1^.

It has been reported that degradation of some organic compounds initially increases the toxicity of the treated solution.^74,75^ This is because some of the DPs are sometimes more toxic than the parent compound. In such cases, the contaminated water could be treated for an extended period of time – to achieve complete degradation of DPs along with target compound – rather than to merely focus the removal of the target compound. To find out whether lindane removal could efficient for the water decontamination or its complete mineralization (degradation of lindane along with its organic products) is inevitable for detoxification of lindane polluted water, the aquatic toxicity of lindane and its detected DPs was determined using the ECOSAR program. The calculated results are depicted in Fig. 4 and Table S5, ESI.† The acute toxicity results showed that the LC_50_ values of lindane, HCB, TCB, DCB, DCP and CBD for fish are 2.24, 0.08, 2.77, 8.52, 25.5 and 232.0 mg L^−1^, respectively. These results indicated that HCB is more toxic than lindane whereas all other products are less toxic than lindane. Generally, the toxicity increases with increasing the number of chlorine atoms in a compound. Thus, the relatively lower toxicity of TCB, DCB, DCP and CBD than lindane could be attributed to the removal of chlorine atoms from these DPs. Since both lindane and HCB possess equal number of chlorine atoms, the higher toxicity of HCB than lindane could be assigned to the aromatic nature of HCB. The acute toxicity of lindane and its detected DPs towards fish follows the order: HCB > lindane > TCB > DCB > DCP > CBD, in agreement with the direct relationship between toxicity and number of chlorine atoms. Similar results – in the same order – were found for daphnia and green algae. In terms of chronic toxicity, lindane and its DPs followed the same order as of acute toxicity (Fig. 4 and Table S5†), except for lindane and TCB toxicity towards green algae, where TCB (ChV = 1.14 mg L^−1^) was a little more toxic than lindane (ChV = 1.15 mg L^−1^). It can be concluded from the toxicity results that lindane degradation by solar/H_2_O_2_/TiO_2_ and solar/PS/TiO_2_ are effective technologies for the detoxification of lindane contaminated waters as most of the DPs are less toxic than lindane except HCB which is produced at initial stages of the reaction but subsequently degraded as the reaction proceeds.

## 4. Conclusions

The occurrence of lindane in the surface water of Peshawar valley (*i.e.*, Peshawar, Charsadda, Nowshera, Mardan and Swabi districts) was investigated. Out of the 75 samples tested (*i.e.*, 15 samples from each district), 13 samples were found to be contaminated with lindane (*i.e.*, 17.3% detection frequency). In most of the contaminated samples, lindane concentration exceeded the maximum acceptable level (MAL) for a single pesticide in surface water, *i.e.*, 1 μg L^−1^, indicating an issue of concern for the environment and living organisms including human beings. The maximum lindane concentration (*i.e.*, 2.60 μg L^−1^) was detected in water sample of Nowshera, attributed to the location of a sealed pesticide factory in this region, established in 1963, but closed down in 1994.

The current study can be extended to other regions in order to get a clear baseline data for the entire country on assessment of lindane and other organochlorine pesticides. The habitants of Amangarh (Nowshera), where the closed pesticide factory is located, must be alarmed on the health hazards associated with lindane contamination. It was concluded that solar light assisted TiO_2_ photocatalysis was an effective method for the removal of lindane from the real water samples. The efficiency of solar/TiO_2_, solar/H_2_O_2_/TiO_2_ and solar/PS/TiO_2_ processes were slightly lowered in natural water samples as compared to Milli-Q water due to the water constituents scavenging effects. However, the photocatalytic degradation pathways of lindane were apparently unaffected by the natural water constituents as similar DPs were detected in natural water sample as in Milli-Q water – detected in our previous studies. The obtained results could be used to design an Integrated Management Program for controlling the concentration of pesticides in the aquatic environment.

## Author contributions

Sanaullah Khan: conceptualization, data curation, formal analysis, investigation, methodology, writing – original draft. Javed Ali Khan: conceptualization, project administration, supervision, funding acquisition, validation, writing – review & editing. Noor S. Shah: conceptualization, data curation, formal analysis, investigation, methodology, resources, writing – original draft. Murtaza Sayed: conceptualization, validation, writing – review & editing. Muhammad Ateeq: software, validation, writing – review & editing. Sabah Ansar: conceptualization, funding acquisition, writing – review & editing. Umar Farooq: conceptualization, writing – review & editing. Grzegorz Boczkaj: conceptualization, writing – review & editing.

## Conflicts of interest

There are no conflicts of interest to declare.

## Supplementary Material

RA-013-D3RA03610C-s001
