# Supplementary material for: Determination of lindane in surface water samples and its degradation by hydrogen peroxide and persulfate assisted TiO2-based photocatalysis
Source: RSC Adv. 2023 Jul 10;13(30):20430–42. doi: 10.1039/d3ra03610c (PMC10331374; doi:10.1039/d3ra03610c)
Supplement: RA-013-D3RA03610C-s001 [file RA-013-D3RA03610C-s001.pdf]

Electronic Supplementary Material (ESI) for RSC Advances.
This journal is © The Royal Society of Chemistry 2023

Determination of lindane in surface water samples and its degradation by hydrogen peroxide and persulfate assisted TiO_2-based photocatalysis

Sanaullah Khan[1], Javed Ali Khan[2,*], Noor S. Shah[3], Murtaza Sayed[4], Muhammad Ateeq[2], Sabah Ansar[5], Grzegorz Boczkaj[6,7], Umar Farooq[8,9]

[1]Departmen of Chemistry, Women University Swabi, 23430, Pakistan

[2]Department of Chemistry, Abdul Wali Khan University Mardan, Mardan 23200, Pakistan

[3]Department of Environmental Sciences, COMSATS University Islamabad, Vehari Campus, 61100, Pakistan

[4]Radiation Chemistry Laboratory, National Centre of Excellence in Physical Chemistry, University of Peshawar, Peshawar 25120, Pakistan

[5]Department of Clinical Laboratory Sciences, College of Applied Medical Sciences, King Saud University, P.O. Box 10219, Riyadh, 11433, Saudi Arabia

[6]Department of Sanitary Engineering, Faculty of Civil and Environmental Engineering, Gdansk University of Technology, G. Narutowicza St. 11/12, 80-233, Gdansk, Poland

[7]EkoTech Center, Gdansk University of Technology, G. Narutowicza St. 11/12, 80-233, Gdansk, Poland

[8]Department of Chemistry, COMSATS University Islamabad, Abbottabad-Campus 22060, Abbottabad, Pakistan

[9]Beijing National Laboratory for Molecular Sciences, State Key Laboratory of Molecular Reaction Dynamics, Institute of Chemistry, Chinese Academy of Sciences, Beijing 100190, China

[*]Corresponding author e-mails: javedkhan@awkum.edu.pk; khanjaved2381@gmail.com (J.A. Khan)

Fax: +92-937-542189; Tel: +92-937-929122

Table S1. Physico-chemical parameters of the water sample collected from Amangarh, Nowshera (N7) where maximum concentration of lindane (2.60 μg L^{-1}) was detected.

pH	Conductivity (μS)	TOC (mg/L)	TH (mg/L)	T. alkalinity (mg/L)	TSS (mg/L)	TDS (mg/L)	Nitrates (mg/L)	Chlorides (mg/L)	Sulphates (mg/L)	Turbidity (NTU)
7.3	712.8	4.58	286.5	172.5	245.6	368.4	3.4	356.3	86.5	17.6

*T (Total); TOC (Total organic carbon); TH (Total Hardness); TTS (Total suspended solids); TDS (Total dissolved solids)

Table S2. Method validation of SPME followed by GC-ECD, in terms of R^2-value, LOD and LOQ.

Analyte	Equation	Correlation coefficient (R^2)	LOD (μg L^{-1})	LOQ (μg L^{-1})
Lindane	y = 66.71 x + 2.27	0.997	0.008	0.037

Table S3. Cultivation area, cotton production, yield value and pesticides consumed in Pakistan during 1997-2006 (Economic Survey of Pakistan; 2003-2004 and 2005-2006).

Year	Area (Thousand hectares)	Production (Thousand bales)	Yield (kg/ha)	Total pesticides in tones (Imported + Locally manufactured)
1997-98	2960	9184	528	42,732.0
1998-99	2923	8790	512	44,872.0
1999-00	2983	11,240	641	45,232.0
2000-01	2927	10,732	570	44,762.0
2001-02	3116	10,613	579	46,132.0
2002-03	2794	10,211	622	46,943.0
2003-04	2989	10,048	572	47,432.0
2004-05	3193	14,265	760	46,572.0
2005-06	3096	12,417	682	46,951.0

Table S4. Effect of different scavengers on k_{obs} of lindane by solar/TiO_2. Experimental conditions: $[Lindane]_0 = 2.60$ μg L^{-1}; $[MeOH]_0 = [i\text{-}PrOH]_0 = [BQ]_0 = 50$ mM; $[PDC]_0 = 100$ μM; Mass of TiO_2 film = 9.02 mg; Thickness of TiO_2 film = 1.02 μm; Area of TiO_2 film = 3750 mm^2; pH = 7.3.

Scavenger	k_{obs} (h^{-1})
Control (No scavenger)	0.0309
Iso-PrOH (*i*-PrOH)	0.0126
Methanol (MeOH)	0.0089
Benzoquinone (BQ)	0.0258
Potassium dichromate (PDC)	0.0230

Table S5. Acute and chronic toxicities of lindane and its detected DPs towards fish, daphnia

Compound	Acute toxicity (mg/L)			Chronic toxicity (mg/L)		
	LC_{50}	LC_{50}	EC_{50}	ChV	ChV	ChV
	Fish	Daphnia	Green Algae	Fish	Daphnia	Green Algae
Lindane	2.24	1.57	2.76	0.280	0.273	1.15
HCB	0.08	0.065	0.211	0.012	0.017	0.122
TCB	2.77	1.88	2.93	0.334	0.301	1.14
DCB	8.52	5.45	6.62	0.958	0.738	2.26
DCP	25.5	15.6	15.8	2.72	1.87	4.87
CBD	232.0	128.0	84.0	21.9	11.5	20.6

and green algae.
